# Transitional care for the highest risk patients: findings of a randomised control study

**DOI:** 10.5334/ijic.2003

**Published:** 2015-10-22

**Authors:** Kheng Hock Lee, Lian Leng Low, John Allen, Sylvaine Barbier, Lee Beng Ng, Matthew Joo Ming Ng, Wei Yi Tay, Shu Yun Tan

**Affiliations:** Department of Family Medicine & Continuing Care, Singapore General Hospital, Singapore and Family Medicine, Duke-NUS Graduate Medical School, Singapore; Department of Family Medicine & Continuing Care, Singapore General Hospital, Singapore and Family Medicine, Duke-NUS Graduate Medical School, Singapore; Centre for Quantitative Medicine, Duke-NUS Graduate Medical School, Singapore; Centre for Quantitative Medicine, Duke-NUS Graduate Medical School, Singapore; Department of Family Medicine & Continuing Care, Singapore General Hospital, Singapore and Family Medicine, Duke-NUS Graduate Medical School, Singapore; Department of Family Medicine & Continuing Care, Singapore General Hospital, Singapore and Family Medicine, Duke-NUS Graduate Medical School, Singapore; Department of Family Medicine & Continuing Care, Singapore General Hospital, Singapore and Family Medicine, Duke-NUS Graduate Medical School, Singapore; Department of Family Medicine & Continuing Care, Singapore General Hospital, Singapore

**Keywords:** readmissions, transitional care, length of stay, acuity of admission, comorbidity of patient, emergency department utilisation score, integrated care

## Abstract

**Background:**

Interventions to prevent readmissions of patients at highest risk have not been rigorously evaluated. We conducted a randomised controlled trial to determine if a post-discharge transitional care programme can reduce readmissions of such patients in Singapore.

**Methods:**

We randomised 840 patients with two or more unscheduled readmissions in the prior 90 days and Length of stay, Acuity of admission, Comorbidity of patient, Emergency department utilisation score ≥10 to the intervention programme (*n* = 419) or control (*n* = 421). Patients allocated to the intervention group received post-discharge surveillance by a multidisciplinary integrated care team and early review in the clinic. The primary outcome was the proportion of patients with at least one unscheduled readmission within 30 days after discharge.

**Results:**

We found no statistically significant reduction in readmissions or emergency department visits in patients on the intervention group compared to usual care. However, patients in the intervention group reported greater patient satisfaction (*p* < 0.001).

**Conclusion:**

Any beneficial effect of interventions initiated after discharge is small for high-risk patients with multiple comorbidity and complex care needs. Future transitional care interventions should focus on providing the entire cycle of care for such patients starting from time of admission to final transition to the primary care setting.

**Trial Registration:**

Clinicaltrials.gov, no NCT02325752

## Introduction

Health care systems around the world are struggling to meet the increasing demand for hospital resources. Ageing populations and the increasing prevalence of chronic diseases and comorbidities exacerbate this rising demand [1]. Rich and poor nations alike find difficulty coping with this crisis caused by rising demand and pressure on finite resources [2]. Transitional care programmes have been proposed as one solution for ameliorating this situation [3]. These are interventional programmes, usually multidisciplinary in nature, aimed at improving health care provider outcomes through improvements in care coordination and continuity for patients in transition between health care settings. The theoretical concept behind transitional care intervention is that readmissions are largely preventable if issues can be addressed that predispose patients to decompensate and return to seek treatment at the hospital. While the concept seems valid on its face and multiple anecdotal descriptive studies are available, there have been few well-designed studies supporting the effectiveness of such programmes, especially for very sick patients with higher risk of death and readmission [4]. The difficulties are mainly due to the multifaceted nature of these complex interventions and the difficulty of performing randomised controlled studies on transitional care programmes. Transitional care programmes have been targeted at patients with specific diagnoses [5, 6] or focused on geriatric adults [7–10] or general medical patients at average risk of readmission [11, 12]. Others have excluded patients who were too complex or too ill [3, 13]. Moreover, there is reason to believe that transitional care interventions developed in the USA and Canada will require further evaluation before implementation in Asian countries due to major differences in health systems structure and funding. With a rapidly ageing population and rising health care utilisation, there is great interest in Singapore to develop effective transitional care programmes and keep long-term health care costs affordable.

In 2010, the all-cause 30-day readmission rate in Singapore was 11.6% and 19% in patients aged 65 years and above [14]. In 2011, the Singapore General Hospital implemented a transitional care programme targeting patients at highest risk of readmission and then evaluated its effectiveness in a randomised controlled study. In the programme, a multidisciplinary team provides post-discharge care through patient monitoring via telephone follow-up, medication reconciliation, early review clinics and home visits. The primary aim was to find out if such a transitional care programme could reduce the rate of unscheduled readmissions. The secondary aim was to determine if the proposed transitional care programme improved emergency department attendance rates and patient satisfaction. Our primary hypothesis was that the proportion of patients with unscheduled readmissions at 30 days after the index discharge would be 10% lower in the intervention group compared to the care-as-usual control group.

## Methods

### Study design

We conducted an open, randomised controlled study from August 2011 to September 2012 at the Singapore General Hospital on patients receiving the transitional care programme versus patients receiving the usual care. Consented patients were randomised in blocks of six and allocated in a 1:1 ratio to either the transitional care intervention or the control group.

### Setting

Singapore is a city state with a resident population of 3.44 million. The vast majority of the resident population receives their health care within the city. The public sector is the main provider of hospital-based care, with 85% of all hospital beds in public sector hospitals. Singapore General Hospital accounts for about one-quarter of the total acute hospital beds in the public sector. Singapore General Hospital is a not-for-profit tertiary hospital with 36 specialist departments, including family medicine. Singapore General Hospital is wholly owned by the Government of Singapore, and with 1597 beds is the largest hospital in the public health care system. With a workforce numbering above 10,000, Singapore General Hospital admits over 1 million patients every year [15].

### Study participants

The study transitional care programme was provided to patients identified through risk stratification as having high risk of readmission. Patients were eligible if they were older than 21 years, had 2 or more unscheduled admissions to selected medical departments in the 90 days prior to study recruitment, and had a Length of stay, Acuity of admission, Comorbidity of patient, Emergency department utilisation score of ≥10. The Length of stay, Acuity of admission, Comorbidity of patient, Emergency department utilisation score is a validated clinical risk-scoring system in Canada [16]. Patients in Singapore with a Length of stay, Acuity of admission, Comorbidity of patient, Emergency department utilisation score ≥10 had a fivefold higher risk of readmission [17]. The Length of stay, Acuity of admission, Comorbidity of patient, Emergency department utilisation score was calculated based on the last admission before recruitment.

Eligible patients were identified using the hospital admission administrative system. The departments included in this study were internal medicine, gastroenterology, respiratory medicine, neurology and endocrinology. These are departments with high readmission rates that agreed to participate in the study programme.

Exclusion criteria ensured collection of complete data and feasibility of patient follow upon discharge. Patients were excluded if they were non-residents, could not be contacted by telephone, did not have a resident address or if they were residing in long-term care facilities.

All study participants provided written informed consent prior to enrollment in the study. If a patient was unable to provide consent, it was obtained from a legal substitute decision maker. Research ethics approval was granted by the Institutional Review Board of Singapore Health Services [CIRB Ref 2011/380/E].

### Randomisation

Randomisation was independently effected and treatment group allocation was concealed via an off-site telephone service managed by a hospital administrator. Blinding of patients and staff was not considered practicable, but this was not regarded as essential given that primary outcomes were to be assessed through analysis of hospital administrative data.

### Intervention

A multidisciplinary integrated care team comprising one attending doctor, one advance practice nurse, four nurses and one medical social worker delivered the transitional care programme. Physical therapists and pharmacists were available upon request by doctors or nurses. Doctors acted as team leaders. All study doctors were family physicians with an average of 4 years working experience in both the hospital and the community setting. Nurses had an average of 2 years working experience and attended in-house training in case management. All clinicians were from the Department of Family Medicine and Continuing Care in Singapore General Hospital [18]. They were rotated into the service and with minimum rotation duration of 1 month. Our transitional care programme focused on four key areas:

1. Post-discharge surveillance of the patient to ensure adherence to care plans
2. Coordination of follow-up visits with specialist care providers
3. Patient education and caregiver coaching
4. Activation of community and social services

Upon recruitment, patients were interviewed and assessed by the team nurse prior to discharge. Intervention started upon discharge from the hospital. Duration of the intervention programme was 3 months. A telephone follow-up was made within 72 hours after discharge to assess the patient's condition and adherence to treatment plan. Home visits were made within 2 weeks after discharge. During the initial home visit, the nurse assessed the patient's medical condition and health literacy, competency of the care-giver, availability of nursing and home care equipment, adequacy of social support, safety of the home environment and adherence to medication. The nurse addressed any identified areas of deficiency, with help from the multidisciplinary team. Referrals were made to activate needed social and financial support services. Each nurse was responsible for an average of 30 patients at any time during the programme.

A multidisciplinary team meeting was conducted in the morning of every working day. During the meeting, each nurse reported on the status of patients under her care. Newly recruited patients were discussed in detail and management plans updated with input from the team. Patients developing acute issues or who had de-stabilised since discharge were given urgent appointments to an early hospital review clinic staffed by attending physicians from the same department. Stable patients were scheduled for review by the team at a subsequent multidisciplinary team meeting. The interval between multidisciplinary team meeting reviews for patients was allowed to increase as their condition improved. Throughout the intervention period, nurses maintained contact with assigned patients via telephone. Nurses were contactable by patients during office hours. Scheduled calls to patients were made once per week, on average.

### Control

Patients in the control group received usual medical care provided by the admitting inpatient team of the hospital. On discharge, patients may be referred to primary care provider, specialists in the outpatient clinic and ambulatory community services as considered necessary by the medical team. Patients receive an abbreviated standardised patient copy of the hospital discharge summary listing their medical diagnoses and medications. For this study, there was no contact between patients in the control group and the study team throughout the 3-month interval. A scheduled telephone call was made at the end of 3 months when patients were invited to participate in a telephone survey.

### Sample size, outcomes and analysis

Sample size was determined based on an estimated 40% readmission within 30 days for the control group and a relative improvement of 25% in readmission for patients enrolled in the intervention group. Power was calculated at 80%, level of significance at 5% and attrition at 10%, resulting in a required a sample size of 420 in each group. The statistical analysis was performed on an intention-to-treat basis.

The primary outcome was the proportion of patients who had at least one unscheduled readmission during the 3-month study period. Unscheduled readmission was defined as any admission from the emergency department. Secondary outcomes were emergency department visits during the study period and patient satisfaction with the intervention programme.

The baseline characteristics were presented as mean ± SD for continuous variables and frequency counts and percentages for categorical variables. For baseline variables, the two-sample *t*-test was used to compare means and the Pearson chi-squared test to compare proportions.

The effect of the intervention was assessed by comparing readmission and emergency department visit rates between study groups using a chi-squared test. Results were summarised as relative risk and absolute risk reduction. Responses of the satisfaction survey were reported and compared between the groups using a two-sample test. Data were analysed using SAS v9.2 (SAS Institute Inc., Cary, NC, USA) and the level of significance was set at 5%.

## Results

**Figure 1.**
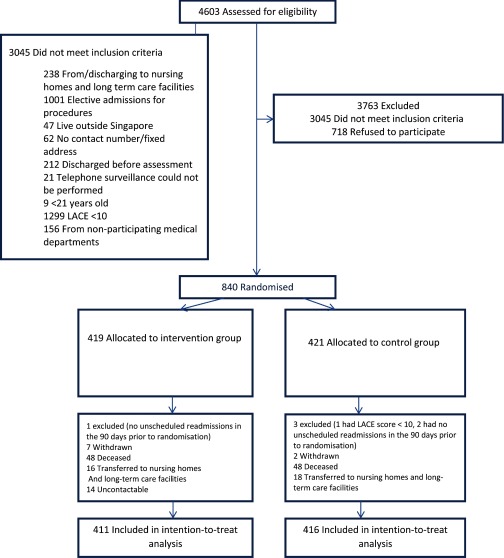
Study flow diagram.

After screening for eligibility, 840 patients were randomised (as shown in Figure 1) with 419 patients allocated to the intervention group and 421 to the control group. Patients who did not meet the study inclusion criteria or withdrew their consent after randomisation were excluded from the final analysis. A total of 411 patients in the intervention group and 416 patients in the control groups were included in an intention-to-treat analysis.

### Baseline characteristics

The characteristics of both study groups were similar at baseline (Table 1). The mean age (SD) of the participants was 68.0 (14.5). The majority of the patients stayed in subsidised ward classes and public housing flats. The average Charlson Comorbidity index and hospital utilisation in the preceding 90 days prior to index admission were similar in both study groups. The mean (SD) Charlson Comorbidity index was 3.4 (1.7). On average, there were 1.41 readmissions, 0.35 emergency visits and 10.59 days of hospitalisation during the 90 days preceding the study.

### Primary outcome

The proportion of patients with at least one unscheduled re-admission within 30 days of intervention was 33.4% in the control group and 28.5% in the intervention group (*p* = 0.124; as shown in Table 2). The relative risk reduction (95% CI) was 0.852 (0.694, 1.045), and the absolute risk reduction (95% CI) was 5.0% (−11.2%, 1.3%). Proportion of patients with readmission within 60 and 90 days after intervention were not statistically significant between the intervention and control groups.

### Secondary outcome

The rate of emergency department visits within 30, 60 and 90 days did not differ significantly between the intervention and control groups.

The intervention group reported a significantly higher level of satisfaction in all aspects of care surveyed (as shown in Table 3): care and concern shown to patients (*p* < 0.001), overall coordination of care (*p* < 0.001), help to better understand the medical condition (*p* < 0.001), understanding of the medical plan (*p* < 0.001) and receipt of help to comply with the recommended treatment plan (*p* < 0.001). Participants in the intervention group also reported higher levels of self-efficacy. Patients in the intervention group reported greater confidence in coping with the illness (*p* < 0.001) and reduced stress and anxiety related to the illness (*p* < 0.001).

## Discussion

Our study indicates that the transitional care programme had a positive trend towards reducing the rate of readmission at 30 days post-discharge, and improving patient satisfaction regarding care. Although results on our primary aim of reducing the readmission rate did not achieve statistical significance at the 0.05 level at 30, 60 or 90 days, a reduction of 5.0 percentage points (from 33.4 to 28.5%, 95% CI:–11.2% to 1.3%) was observed at the 30-day time point (*p* = 0.124). The study was designed to test the primary hypothesis of a 10% difference in unscheduled readmissions between the groups. It was under-powered to detect a difference of 5%, which may still be a clinically important reduction as it translates to a 14.8% reduction in readmission rates in a high-risk patient group. This paper is the first to show that a care transition programme in Singapore targeted at discharged patients is feasible and has a positive impact on patient satisfaction and suggestive evidence on reduced health care utilisation.

Our target population was the select sub-group of patients with the highest risk of readmission and death among all patients, which stands in contrast to previously reported successful interventions in less challenging patient populations [7, 9, 11, 19]. Of 4603 patients who met the first selection criterion of two or more unscheduled readmissions in 90 days, 1299 were excluded due to lower risk based on a Length of stay, Acuity of admission, Comorbidity of patient, Emergency department utilisation score of <10.

Patients were included in our study even if they were non-English speaking or had cognitive impairment. By 30 days, about a third of patients in the control arm had been readmitted at least once. This observed rate was much higher than that reported for local patients aged 65 years and above [14], or in other interventional trials [7, 9, 11, 19, 20]. By 90 days, more than half of the patients in both arms had had at least 1 readmission. It could be anticipated that in such a population with chronic illness, high comorbidity and complex needs, any interventional effect to reduce readmissions would likely be small. However, there may be improvements in quality of life outcomes that we did not evaluate in our study. Future studies should consider other outcome measures on Health-Related Quality of Life such as Medical Outcomes Short Study Form Health Survey and the quality of care transitions such as the Care Transitions Measure 15 in the evaluation of transitional care programmes. To the extent our findings are generalisable, transitional programmes that target only patients at the highest end of risk spectrum are likely to show only small improvements in readmission outcomes. In such patient populations, hospitalisation episodes are likely to be more costly than those for lower risk patients. To determine whether a small difference in outcome produced by a transitional care programme is cost-effective would require further evaluation. Conversely, transitional care programmes directed towards medium- or low-risk patients may produce more impressive results.

In our transitional care programme, the intervention began after the patient was discharged from the hospital. While we cannot infer the effectiveness of additional pre-discharge interventions [7, 9, 11, 19, 21], as our study population differs from previous trials, it is possible that upstream interventional studies could show greater benefit in support of effectiveness of transitional care programmes for higher risk patients [22]. Ensuring transition of care to a primary care provider was distinctly lacking in all the interventional studies reported to date [22, 23]. Future research in this area should perhaps focus on providing transitional care within the entire cycle of care for such patients—from time of admission to final transition to the primary care setting. The results of such studies would be helpful towards increasing our understanding of how we can optimise the benefits of transitional care and develop better strategies of patient risk stratification.

Better understanding of the value of transitional care programmes can be gained by investigating differences in outcome in relation to cost of achieving them. Studies of cost-effectiveness of complex interventions such as transitional care are fraught with difficulties. Attempts to study cost-effectiveness have often been compromised by underlying broad assumptions regarding what is charged to the patient or the third party payer rather than the true cost of care [24]. This approach makes it difficult to study cost-effectiveness as it does not represent the true cost of providing care nor reflect the true cost of care. Perhaps a better understanding of the cost-effectiveness of transitional care programmes could be achieved by using activity-based costing [25]. Transitional care holds great potential as a solution for increasing efficiency in the use of limited health care resources. The capacity to deliver transitional care is limited and should therefore be provided to segments of the patient population where it is most likely to provide the greatest benefit. Improvement in outcome at the same unit cost for low-risk patients may not provide the same value as similar improvement achieved for higher risk patients. We need to work towards identifying segments of the patient population that will yield the highest return on investment for transitional care. We will also need to continue work on improving our understanding of how transitional care can be improved by studying the different models of delivery and distilling the reasons for success or failure.

## Limitations

Our trial could not be blinded; however, it is unlikely that this had any effect on our findings as the outcomes were objective and extracted from the hospital administrative database. We acknowledge potential bias in the patient satisfaction survey as the assessors were not blinded to whether the patient was in the control or intervention group.

We delivered our service to the population of patients considered to have the greatest needs, with high levels of comorbidities and care complexity. This limits somewhat the generalisability of our findings, especially for transitional care programmes with different selection criteria. As we are a tertiary care hospital, our patients came from clinical departments with high rates of unscheduled readmissions. The result of this study may not be generalisable to patients admitted to other clinical departments and hospitals with different case mixes.

## Conclusion

Our findings provide initial evidence that patients at the highest risk for readmission may benefit from transitional care programme. Patient satisfaction may be substantially improved. For better outcome to be achieved the intervention in such programmes may need to extend the intervention to include the full of cycle of care.

## Figures and Tables

**Table 1.**
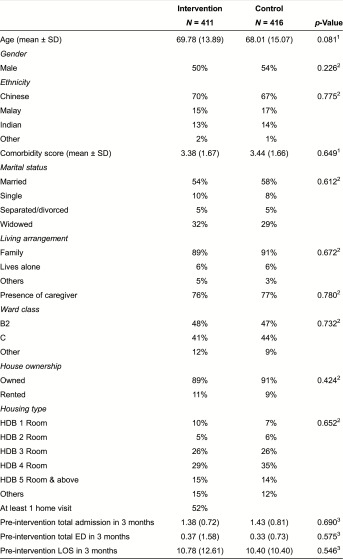
Baseline characteristic of participants

^1^Student's *t*-test; ^2^Chi-squared; ^3^Negative binomial regression.

**Table 2.**
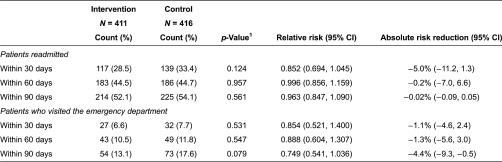
Rate of readmission and emergency department visit

^1^Chi-squared test.

**Table 3.**
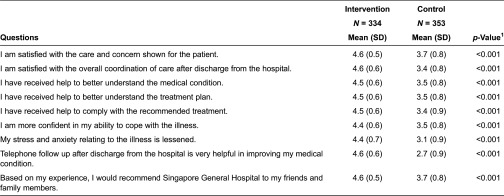
Patient satisfaction survey

^1^Two-sample Student's *t*-test.
